
JOURNAL INFORMATION
==============================
NLM Title Abbreviation: Eur J Gen Pract
Journal ID: Eur J Gen Pract
NLM Journal ID: 9513566

The European Journal of General Practice

ISSN: 1381-4788
EISSN: 1751-1402
Publisher: Taylor & Francis

ARTICLE INFORMATION
==============================
PMCID: PMC9970190
DOI: 10.1080/13814788.2023.2171393
Article ID: 2171393
Article version: 1
Article version: Version of Record
Subjects: Abstract, Abstracts

Fostering clinical research in general practice and family medicine. Selected abstracts from the 93rd EGPRN conference Halle, Germany, 14–17 October 2021

All abstracts of the conference can be found at the EGPRN website https://www.egprn.org/page/conference-abstracts

Abstracts

European Journal of General Practice

Electronic publication date: 2023 Feb 23
Publication date: 2023
Volume: 29
Issue: 1
Electronic Location ID: 2171393
Copyright: © 2023 The Author(s). Published by Informa UK Limited, trading as Taylor & Francis Group.
Copyright year: 2023
Copyright holder: The Author(s)
License: This is an Open Access article distributed under the terms of the Creative Commons Attribution License (http://creativecommons.org/licenses/by/4.0/), which permits unrestricted use, distribution, and reproduction in any medium, provided the original work is properly cited.
License URL: https://creativecommons.org/licenses/by/4.0/

Figure count: 0
Table count: 0
Page count: 11

==============================


 

Keywords: COVID-19, patient centred care, mental health, clinical topics

Introduction to the theme of the conference: Fostering clinical research in general practice and family medicine

Subjects: Abstract, Keynote Lectures

International keynote lecture: How do we integrate clinical research in general practice? It is time to wake the sleeping beauty.

Heymann Anythony
The Department of Family Medicine, The Sackler School of Medicine, University of Tel Aviv , Tel Aviv , Israel

Subjects: Abstract, Keynote Lectures

National keynote lecture: Equipped for the future? Disruptive innovation – data – studies

Franken Christian
DiHeSys Digital Health Systems , Schwäbisch Gmünd , Germany

Subjects: Abstract, Poster Prize Winner

Experiences and expectations of medical students and GP teachers regarding long-term mentoring relationships in longitudinal general practice tracks: Preliminary results of an ongoing qualitative study

Scholz Anna
Gehres Vera
Schrimpf Anne
Bleckwenn Markus
Deutsch Tobias
Geier Anne-Kathrin
Department of General Practice, Faculty of Medicine, University of Leipzig , Leipzig , Germany
CONTACT anna.scholz@medizin.uni-leipzig.de
Keywords: General practice track, medical students, mentoring, expectations, qualitative design

Subjects: Abstract, Selected Abstracts

BNT162b2 vaccine effectiveness in preventing asymptomatic infection with SARS-CoV-2 virus: A nationwide historical cohort study

Zacay Galia
Shasha David
Bareket Ronen
Kadim Itai
Hershkowitz-Sikron Fabienne
Tsamir Judith
Mossinson David
Heymann Anthony
Meuhedet HMO , Ganei-Tikva , Israel
CONTACT gzacay@hotmail.com
Keywords: Covid-19, SARS-CoV-2, vaccine effectiveness, asymptomatic infection, observational study

Subjects: Abstract, Poster Prize Winner

The COVID-19 pandemic: Reorganisation of health services and coping of health care workers in primary healthcare

Outeirinho Conceição
Braga Raquel
Costa-Gomes Joana
Alves Luís
Margarida Cruz Ana
Clavería Ana
Núñez-Torrón Antón
Jotic Marina
Dermutas Jacopo
Çalışkan Tugba
Tkachenko Victoria
Jorge Poliana
ICBAS-UP, SNS Portugal , Porto , Portugal
CONTACT couteirinho@gmail.com
Keywords: Primary Health Care, COVID-19, SARS-CoV-2, pandemic, health care services, primary care management, anxiety, emergency management

Subjects: Abstract, Poster Prize Winner

Benefits and challenges of using virtual primary care during the COVID-19 pandemic: From key lessons to a framework for implementation

Li Edmond
Tsopra Rosy
Gimenez Geronimo Larrain
Serafini Alice
Gusso Gustavo
Lingner Heidrun
Fernandez Maria Jose
Irving Greg
Petek Davorina
Hoffmann Robert
Lazic Vanja
Ensieh Memarian
Koskela Tuomas
Collins Claire
Espitia Sandra Milena
Clavería Ana
Nessler Katarzyna
O’neill Braden Gregory
Hoedebecke Kyle
Ungan Mehmet
Laranjo Liliana
Ghafur Saira
Fontana Gianluca
Majeed Azeem
Car Josip
Darzi Ara
Neves Ana Luisa
Center for Health Technology and Services Research, University of Porto , Porto , Portugal
CONTACT ana.luisa.neves@gmail.com
Keywords: Primary care, telemedicine, digital health

Subjects: Abstract, Poster Prize Winner

The long-term effect of COVID-19 – Primary care survey

Vinker Shlomo
Cohen Avivit Golan
Green Ilan
Merzon Eugene
Israel Ariel Yehuda
Abramovich Eva
Itamary Hagit
Family Medicine, Leumit Health Services , Ashdod , Israel
CONTACT vinker01@gmail.com
Keywords: COVID-19, long COVID, primary care

Subjects: Abstract, Poster Prize Winner

A study of burnout and associated factors in Irish GPs and GP trainees during the COVID-19 pandemic

Mc Kenna Darragh
Raghoonath Mala
Abioye Imoleayo
Abdoul-Rahman Leila
Collins Claire
South West Training Programme of the Irish College of General Practitioners, Munster Technological University , Tralee , Ireland
CONTACT darraghmckenna14@gmail.com
Keywords: Exhaustion, disengagement, burnout, OLBI, Oldenburg burnout inventory, wellbeing, COVID-19, pandemic, Irish, Ireland, Europe, European, general practice, primary care, GP, trainee, training

Subjects: Abstract, Patient Centred Care

Challenges of research on person-centred care in general practice: A scoping review

Burgers Jako
Van Der Weijden Trudy
Bischoff Erik
Dutch College of General Practitioners , Utrecht , The Netherlands
CONTACT j.burgers@nhg.org
Keywords: General practice, family practice, patient-centred care, patient outcome assessment, scoping review

Subjects: Abstract, Patient Centred Care

The patient-centred care and its relation to the outcomes of care in family medicine

Petricek Goranka
Bajkovic Mia
Vuletic Gorka
Cerovecki Venija
Ozvacic Adzic Zlata
Hanzevacki Miroslav
University of Zagreb School of Medicine , Zagreb , Croatia
CONTACT goranka.petricek@mef.hr
Keywords: Patient-centered care, outcomes of care, family medicine

Subjects: Abstract, Patient Centred Care

Shared decision-making enhances vaccination rates in adult patients in outpatient care – A systematic review and meta-analysis

Sanftenberg Linda
Kuehne Flora
Anraad Charlotte
Jung-Sievers Caroline
Dreischulte Tobias
Gensichen Jochen
Institute of General Practice and Family Medicine , Munich , Germany
CONTACT linda.sanftenberg@med.uni-muenchen.de
Keywords: Shared decision making, vaccination, influenza, pneumococcal disease, systematic review, adult

Subjects: Abstract, Patient Centred Care

Impact of multimorbidity on healthcare professional task-shifting potential in patients with type 2 diabetes in primary care: A French cross-sectional study

Supper Irene
Bourgueil Yann
Ecochard René
Letrilliart Laurent
Department of General Practice Lyon , Lyon , France
CONTACT irenesupper@hotmail.com
Keywords: Interprofessionnal collaboration, diabetes, multimorbidity, cross-sectional study

Subjects: Abstract, Mental Health

Experiences of patients with common mental disorders concerning team-based primary care and a person-centered dialogue meeting: An intervention to promote return to work

Saxvik Ausra a
Törnbom Karin b
a Primary Health Care/Department of Public Health and Community Medicine, Institute of Medicine, Sahlgrenska Academy, University of Gothenburg , Gothenburg , Sweden
b Department of Social Work, University of Gothenburg , Hono , Sweden
CONTACT ausra.saxvik@gmail.com
Keywords: Common mental disorders, rehabilitation, collaboration, qualitative study, person-centred dialogue meeting, return to work

Subjects: Abstract, Mental Health

Evaluation of a pragmatic c-RCT to discontinue (z)BZD use for insomnia in general practice

Coteur Kristien
Matheï Catharina
Anthierens Sibyl
Van Den Broeck Kris
Van Nuland Marc
KU Leuven , Leuven , Belgium
CONTACT kristien.coteur@kuleuven.be

Subjects: Abstract, Mental Health

‘Never change a winning team:’ GPs’ perspectives on discontinuation of long-term antidepressants

Van Leeuwen Ellen
Anthierens Sibyl
Van Driel Mieke
Sutter An De
Vandenbranden Evelien
Christiaens Thierry
University of Ghent , Ghent , Belgium
CONTACT ellen.vanleeuwen@ugent.be
Keywords: Long-term antidepressants, discontinuation, general practitioner, depressive disorder, anxiety disorder, qualitative review

Subjects: Abstract, Mental Health

Clinical outcome data of first cohort of chronic pain patients treated with cannabis-based sublingual oils in the United Kingdom – Analysis from the UK Medical Cannabis Registry

Kawka Michal
Erridge Simon
Holvey Carl
Coomber Ross
Usmani Azfer
Sajad Mohammad
Platt Michael W.
Rucker James J.
Sodergren Mikael H.
Imperial College Medical Cannabis Research Group , London , United Kingdom
CONTACT mik17@ic.ac.uk
Keywords: Cannabinoids, medical cannabis, health-related quality-of-life, chronic pain

Subjects: Abstract, Clinical Topics

Heartwatch: A chronic disease management programme for heart disease in Ireland

Stanley Fintan
Homeniuk Robyn
Collins Claire
Irish College of General Practitioners , Dublin , Ireland
CONTACT fintan.stanley@icgp.ie
Keywords: Chronic disease management, cardiovascular, heart disease

Subjects: Abstract, Clinical Topics

Factors that influence the mortality rate among individuals who are over 65 years old and utilise home health care services

Ayhan Nida
Unalan Pemra C.
Korkmaz Büşra Çimen
Department of Family Medicine, School of Medicine, Marmara University , Istanbul , Turkey
CONTACT pcunalan@gmail.com
Keywords: Elderly, mortality, home health care services

Subjects: Abstract, Clinical Topics

Men’s knowledge about erectile dysfunction and its management options: Results from primary care study in Latvia

Pankova Kamila
Silina Vija
Riga Stradins University , Riga , Latvia
CONTACT kamila.kerimova78@gmail.com
Keywords: Latvia, men, erectile disfunction, family doctor

Subjects: Abstract, Clinical Topics

Rapid detection of NAFLD and its evolutionary stages toward cirrhosis at a targeted population: Multiparametric Liver Ultrasonographic Screening (MLUS) and artificial intelligence with fibrosis risk stratification by family physicians

Iacob Mihai
Remes Ana
Stoican Madalina
The European Ultrasound Working Group/Euvekus/Eadus, 2. Timis Society of Family Physicians, 3. Timis Medical Chamber , Timisoara , Romania
CONTACT dr_iacob@yahoo.com
Keywords: Multiparametric liver ultrasound (MLUS), NAFLD screening in primary healthcare, morphometric-ultrasound, APRI Score, artificial intelligence, point of care ultrasonography (POCUS)

